# Registered Reports: A new chapter at *Ecology & Evolution*

**DOI:** 10.1002/ece3.10023

**Published:** 2023-04-27

**Authors:** Ragnhild Gya, Kristine Birkeli, Ingrid J. Dahle, Christopher G. Foote, Sonya R. Geange, Joshua S. Lynn, Joachim P. Töpper, Vigdis Vandvik, Camilla Zernichow, Gareth B. Jenkins

**Affiliations:** ^1^ Department of Biological Sciences University of Bergen Bergen Norway; ^2^ Bjerknes Center for Climate Research Bergen Norway; ^3^ John Wiley & Sons Ltd Oxford UK; ^4^ Department of Earth and Environmental Sciences The University of Manchester Manchester UK; ^5^ Norwegian Institute for Nature Research Bergen Norway

## Abstract

Ecology & Evolution has published its first Registered Report and offers the perspective of the editor, author, and student on the publication process.

## 1 THE EDITOR'S PERSPECTIVE

*Ecology & Evolution* has been, since launch, a journal that strives to publish high‐quality research, but places no emphasis on the perceived “novelty” or potential “impact” of the results we publish. Instead, the goal is to serve the ecology and evolution communities and provide an outlet for sound research and communication without acting as arbitrary gatekeepers. We strive to be experimental. This year, we have published the first manuscripts of a new type of article for *Ecology & Evolution*—Registered Reports.

The approach to publishing this article type sits particularly well with our sound science philosophy. Registered Reports are well‐described in the literature (e.g., Chambers et al., 2014), but in brief, they are essentially a research article that is published in two stages. In Stage 1, the authors submit the Introduction and Methods of the paper, thereby providing a detailed outline of the motivation and proposed methods and analyses, which are peer‐reviewed before any data collection/analysis takes place and accepted “in principle” with a commitment to publish if the authors adhere to the agreed outline. The authors must register the approved protocol on open platforms such as Open Science Framework (https://osf.io/) or Authorea (https://www.authorea.com/). Then, the actual research, as described in the “in principle” accepted Stage 1 report, is conducted. Stage 2 is the “complete” article (i.e., including Results/Discussion), which is ideally reviewed by the same referees to ensure that the research approved in stage 1 has been appropriately conducted and reported. Further details on what is required can be found here (https://www.cos.io/initiatives/registered‐reports).

Registered Reports first saw the light of day in 2013 (Chambers, 2013), with early adoption among a number of psychology journals (Chambers & Tzavella, 2022). Registered Reports are now increasingly being adopted across a range of STEM journals (e.g., Hardwicke & Ioannidis, 2018; Kiyonaga & Scimeca, 2019; Nosek & Lakens, 2014; https://www.nature.com/articles/d41586‐023‐00506‐2), so at this point, they are certainly not novel, but the journey to widespread usage among ecologists and evolutionary biologists is still in the early stages, with the first journal in the eco‐evo space only adopting them in 2017 (Foote, 2017). *Ecology & Evolution* has now published its first Registered Report (Gya et al., 2023), and the second is in the pipeline (Birkeli et al., 2023). As a journal, we are keen to see more Registered Reports in our field, given their numerous benefits and potential to eliminate many questionable research practices. Specifically, this publication model ensures a reduced emphasis placed on the novelty/impact of the results (a cause close to our hearts at *Ecology & Evolution),* publication of confirmatory/negative results (both of which are vital to reproducibility), a guard against HARKing and p‐hacking (see Fraser et al., 2018), and a reduction in inadvertently poor experimental designs (which can lead to serious flaws such as pseudoreplication). In short, they lead to more robust science, with relatively few costs that can largely be mitigated. We believe that Registered Reports will be an invaluable part of the toolbox to make research more reproducible and open in future, while reducing research waste. This publication format can also be a useful component in teaching and learning of sound academic practices.

Below we discuss both the advantages and disadvantages Registered Reports can bring, by offering a perspective on (a) the publication process of Gya et al. (2023) and (b) using Registered Reports as an educational tool. We hope to see more of this interesting article type in Ecology & Evolution and encourage researchers to get in touch to learn more.

## 2 THE AUTHOR'S PERSPECTIVE

The idea behind the first Registered Report published in *Ecology & Evolution* (Gya et al., 2023) was born from an unexpected opportunity—during summer, our team had collected an excess of seeds from global change experiments in Western Norway (see Vandvik et al., 2020) and were in search of a good use for them. A changing climate results in more extreme climate events, including droughts even in wetter alpine regions. The team thus set out to test whether seeds of alpine plants from drier regions and habitats were better adapted to germinate under drought conditions than populations from wetter habitats. We had heard of Registered Reports, and liked the idea, for all the reasons outlined above. In our case, the Registered Report format offered an opportunity for a very structured and well‐documented experimental planning process, all while seeds were still being cold‐stratified, and so, we decided to give it a go.

A couple of weeks into the labor‐intensive lab experiment, COVID‐19 hit. As this was an experiment that was crucial for a PhD thesis, but also because we already had the Stage 1 acceptance from *Ecology & Evolution* (and hence a guaranteed publication regardless of the outcome of the experiments), our department granted us access to the lab during lockdown, saving the experiment. Once the data were in, “only” results and discussion remained, with no need for “creative” storytelling or adjustments because of lab failures or unexpected results.

In Gya et al. (2023), we expected drought adaptations in germination and seedling traits to be stronger in the driest population of both alpine species compared with the populations from wetter habitats. As we finalized the experiment, we saw that one species responded exactly as we had predicted, while the other did not. If we had not already had an acceptance on our Stage 1 Registered Report, we might have been tempted to exclude the species that did not behave according to expectations from this specific paper, for the sake of a cleaner story and less post‐hoc explanations/caveats. Instead, we had to think more carefully about our results and found an ecological explanation for this discrepancy that might stimulate and guide future research (Gya et al., 2023). We know that large amounts of data are not published because of negative results, or because authors or journals prioritize a simpler and “sexier” storyline (Purgar et al., 2022). By adherence to the Registered Report format, more of that science will be published, which may help correct positive result biases in the literature. Hence, Registered Reports could be a tool to reduce the “research waste” that occurs when “failed” experiments or negative results are not reported, which causes other teams to repeat similar experiments unaware of previous attempts and lessons learned (Purgar et al., 2022).

There are, however, disadvantages to this way of publishing, with an obvious case being unexpected, yet interesting, results that were not anticipated in the original Stage 1. Fortunately, there are contingencies for this. The Registered Report format allows you to include “additional analyses” in a separate section of your results, but these results cannot be the main findings from your study. If unexpected findings really are a breakthrough and deserve to be the main story, you can retract the Stage 1 report and submit the new analysis and storyline as a traditional paper, to either the same or an alternate journal.

Besides “additional analyses” accounting for unplanned inspiration, one concern we had was that the rigid structure of a Registered Report may hinder our ability to adapt the analyses to unexpected data, e.g., if the distribution of data did not meet our prior expectation or if a proposed model turned out to be ill‐suited to the data. However, we found this format nimble enough to account for such unforeseen issues, while keeping the analyses honest by requiring an explanation of such alterations.

Another potential disadvantage is that writing a compelling Introduction can be difficult while you are still “blind” to the results. However, our experience is that this can be used to an advantage, helping focus the Introduction on the key question(s), without having to juggle back and forth between Introduction and Discussion, as is often the case in more standard article‐writing formats.

Finally, delays may occur between submission/approval of Stage 1 and the actual experimental work (e.g., field seasons, live material complications, study program duration); therefore, Registered Reports require a relatively rapid peer‐review process, at least on the Stage 1 submission. It also requires clarifying to Stage 2 reviewers that the Introduction and Methods are already assessed and cannot be changed.

Ultimately, we had a positive experience with our reviewers on this paper, and it did not feel like we were limited by our fixed Introduction or Methods from the accepted Stage 1 Registered Report.

## 3 THE STUDENT'S PERSPECTIVE

Gya et al. (2023) was one of the papers from Gya's PhD. Submitting Stage 1 before any data was collected allowed her early experience with the nuts and bolts of the scientific publication process. Much of the science that never gets published is research from MSc or PhD theses (Purgar et al., 2022), and submitting a Registered Report could help in motivating the student, or other team members, to finalize the paper after leaving the lab.

The lab work in Gya et al. (2023) involved several research internship students, and their involvement in commenting on drafts benefited their understanding of the research and more generally the scientific publication process while helping simplify and clarify the writing. These students expressed that they were much more confident in the data collection because the method was so well‐planned and had been “approved” by expert reviewers.

The Gya et al. (2023) experience inspired several additional Registered Reports in our lab (https://betweenthefjords.w.uib.no/home/), and this time around, the lead authors were MSc students (e.g., Birkeli et al., 2023). Again, we found that the stringent format, the requirement for high‐quality writing at an early stage, and the experience with the “real” review process during the early stage of the students' first independent research experience offered many advantages. The necessity of an early start supports the MSc students' time management and efficiency. As the detailed methodological planning is done at Stage 1, the MSc students felt more confident about their data collection in the field or in the lab. The experience of MSc students and supervisors working together on the reviewer comments gave the students confidence in being able to defend their thesis, as well as a learning experience for the supervisors. For these reasons, Registered Reports (or simulated versions within university courses) have been suggested as a means for MSc and PhD students to learn about the scientific method and open science (Geange et al., 2021). Doing real science, and being treated as a real scientist by the journals, is empowering for students and supports their self‐efficacy.

Care needs to be taken when considering adopting a Registered Report format for MSc theses, to manage the additional potential stressors associated with the review process and potential time constraints for study redesign. By going through the peer‐review process, the students are set up for real failures, in the form of (potentially harsh) reviewers’ comments and a potential rejection or request for major revisions by a scientific journal. This introduces high stakes into the student's thesis experience, which may be especially problematic for minority or underprivileged background students (Silbiger & Stubler, 2019). Mitigating this requires earnest communication between supervisors and students about potential outcomes, before the decision to submit a Registered Report. Communication is thus key to allowing students the benefits of doing real science early on via the Registered Report format while helping them deal with stress caused by high stakes and potential setbacks. Additionally, the student and the supervisor need to have planned enough time for the potential study redesign that could happen as a result of the peer‐review process at Stage 1 for a Registered Report.

But really, the worst possible outcome is for the first author, be they students or researchers, to have written as well as they can, early on, receive external peer‐review advice on the concept and methods, learn about scientific publishing, and then possibly not being published before the work is completed. It should be remembered that, just like other work, a rejected Registered Report may still be submitted and published as a standard paper later. In this sense, the worst possible outcome of a Registered Report experience does not sound too bad, does it?

## AUTHOR CONTRIBUTIONS

**Ragnhild Gya:** Conceptualization (supporting); writing – original draft (lead); writing – review and editing (lead). **Kristine Birkeli:** Writing – original draft (supporting); writing – review and editing (supporting). **Ingrid J. Dahle:** Writing – original draft (supporting); writing – review and editing (supporting). **Christopher Foote:** Writing – original draft (supporting); writing – review and editing (supporting). **Sonya R. Geange:** Writing – original draft (supporting); writing – review and editing (supporting). **Joshua S. Lynn:** Writing – original draft (supporting); writing – review and editing (supporting). **Joachim P. Töpper:** Writing – original draft (supporting); writing – review and editing (supporting). **Vigdis Vandvik:** Writing – original draft (supporting); writing – review and editing (supporting). **Camilla Zernichow:** Writing – original draft (supporting); writing – review and editing (supporting). **Gareth B. Jenkins:** Conceptualization (lead); writing – original draft (supporting); writing – review and editing (supporting).

## ACKNOWLEDGMENTS

We thank Allen Moore for constructive comments on an earlier draft of this manuscript.

## Data Availability

Data sharing is not applicable to this article as no new data were created or analysed in this study.

## References

[ece310023-bib-0001] Birkeli, K. , Gya, R. , Haugum, S. , Velle, L. G. , & Vandvik, V. (2023). How resilient is Calluna vulgaris to drought during germination and its seedling stage? (in press). 10.1002/ece3.10199 PMC10318425 37408632

[ece310023-bib-0002] Chambers, C. D. (2013). Registered Reports: A new publishing initiative at cortex. Cortex, 49, 609–610. 10.1016/j.cortex.2012.12.016 23347556

[ece310023-bib-0003] Chambers, C. D. , & Tzavella, L. (2022). The past, present and future of Registered Reports. Nature Human Behaviour, 6, 29–42. 10.1038/s41562-021-01193-7 34782730

[ece310023-bib-0004] Chambers, C. D. , Feredoes, E. , Muthukumaraswamy, S. D. , & Etchells, P. J. (2014). Instead of “playing the game” it is time to change the rules: Registered Reports at AIMS neuroscience and beyond. AIMS Neuroscience, 1(1), 4–17. 10.3934/Neuroscience.2014.1.4

[ece310023-bib-0005] Foote, C. F. (2017). BMC ecology is now accepting Registered Reports! BMC Series Blog. https://blogs.biomedcentral.com/bmcseriesblog/2017/08/24/bmc‐ecology‐is‐now‐accepting‐registered‐reports/

[ece310023-bib-0006] Fraser, H. , Parker, T. , Nakagawa, S. , Barnett, A. , & Fidler, F. (2018). Questionable research practices in ecology and evolution. PLoS One, 13(7), e0200303. 10.1371/journal.pone.0200303 30011289 PMC6047784

[ece310023-bib-0007] Geange, S. R. , von Oppen, J. , Strydom, T. , Boakye, M. , Gauthier, T. J. , Gya, R. , Halbritter, A. H. , Jessup, L. H. , Middleton, S. L. , Navarro, J. , Pierfederici, M. E. , Chacón‐Labella, J. , Cotner, S. , Farfan‐Rios, W. , Maitner, B. S. , Michaletz, S. T. , Telford, R. J. , Enquist, B. J. , & Vandvik, V. (2021). Next‐generation field courses: Integrating Open Science and online learning. Ecology and Evolution, 11, 3577–3587. 10.1002/ece3.7009 33898010 PMC8057340

[ece310023-bib-0008] Gya, R. , Geange, S. R. , Lynn, J. S. , Töpper, J. P. , Wallevik, Ø. , Zernichow, C. , & Vandvik, V. (2023). A test of local adaptation to drought in germination and seedling traits in populations of two alpine forbs across a 2000 mm/year precipitation gradient. Ecology and Evolution, 13, e9772. 10.1002/ece3.9772 36778839 PMC9905427

[ece310023-bib-0009] Hardwicke, T. E. , & Ioannidis, J. P. A. (2018). Mapping the universe of registered reports. Nature Human Behaviour, 2, 793–796. 10.1038/s41562-018-0444-y 31558810

[ece310023-bib-0010] Kiyonaga, A. , & Scimeca, J. M. (2019). Practical considerations for navigating registered reports. Trends in Neurosciences, 42(9), 568–572. 10.1016/j.tins.2019.07.003 31470913 PMC9380892

[ece310023-bib-0011] Nosek, B. A. , & Lakens, D. (2014). Registered reports: A method to increase the credibility of published results. Social Psychology, 45(3), 137–141. 10.1027/1864-9335/a000192

[ece310023-bib-0012] Purgar, M. , Klanjscek, T. , & Culina, A. (2022). Quantifying research waste in ecology. Nature Ecology Evolution, 6(9), 1390–1397. 10.1038/s41559-022-01820-0 35864230

[ece310023-bib-0013] Silbiger, N. H. , & Stubler, A. D. (2019). Unprofessional peer reviews disproportionately harm underrepresented groups in STEM. PeerJ, 7, e8247. 10.7717/peerj.8247 31844596 PMC6911688

[ece310023-bib-0014] Vandvik, V. , Skarpaas, O. , Klanderud, K. , Telford, R. J. , Halbritter, A. H. , & Goldberg, D. E. (2020). Biotic rescaling reveals importance of species interactions for variation in biodiversity responses to climate change. Proceedings of the National Academy of Sciences of the United States of America, 117(37), 22858–22865. 10.1073/pnas.2003377117 32868426 PMC7502702

